# Determinants of Successful Weight Loss After Using a Commercial Web-Based Weight Reduction Program for Six Months: Cohort Study

**DOI:** 10.2196/jmir.2648

**Published:** 2013-10-14

**Authors:** Elisa Postrach, Rosa Aspalter, Ulf Elbelt, Michael Koller, Rita Longin, Jörg-Dieter Schulzke, Luzia Valentini

**Affiliations:** ^1^The Division of Nutritional MedicineDepartment of Gastroenterology and HepatologyCharité- Universitätsmedizin BerlinBerlinGermany; ^2^KiloCoach e.U.ViennaAustria; ^3^Department of Endocrinology, Diabetes and NutritionCharité- Universitätsmedizin BerlinBerlinGermany; ^4^Centre for Clinical StudiesUniversity Hospital RegensburgRegensburgGermany

**Keywords:** Internet, weight loss, overweight, obesity, weight reduction program, efficiency, program evaluation, preventive health services, sex

## Abstract

**Background:**

The Internet is widely available and commonly used for health information; therefore, Web-based weight loss programs could provide support to large parts of the population in self-guided weight loss. Previous studies showed that Web-based weight loss interventions can be effective, depending on the quality of the program. The most effective program tools are visual progress charts or tools for the self-monitoring of weight, diet, and exercises. KiloCoach, a commercial program currently available in German-speaking countries, incorporates these features. A previous investigation showed that the program effectively supports users in losing weight.

**Objective:**

We investigated weight loss dynamics stratified by weight loss success after 6-month use of KiloCoach. Furthermore, we analyzed possible associations between intensity of program use and weight loss. The results are intended for tailoring user recommendations for weight-loss Internet platforms.

**Methods:**

Datasets of KiloCoach users (January 1, 2008 to December 31, 2011) who actively used the platform for 6 months or more were assigned to this retrospective analysis. Users (N=479) were 42.2% men, mean age of 44.0 years (SD 11.7), with a mean body mass index (BMI) of 31.7 kg/m^2^ (SD 3.2). Based on the weight loss achieved after 6 months, 3 success groups were generated. The unsuccessful group lost <5%, the moderate success group lost 5%-9.9%, and the high success group lost ≥10% of their baseline body weight. At baseline, the unsuccessful (n=261, 54.5%), moderate success (n=133, 27.8%), and high success (n=85, 17.8%) groups were similar in age, weight, BMI, and gender distribution.

**Results:**

After 6 months, the unsuccessful group lost 1.2% (SD 2.4), the moderate success group lost 7.4% (SD 1.5), and the high success group lost 14.2% (SD 3.8) of their initial weight (*P*<.001). Multivariate regression showed that early weight loss (weeks 3-4), the total number of dietary protocols, and the total number of weight entries were independent predictors for 6-month weight reduction (all *P*<.001) explaining 52% of the variance in weight reduction. Sensitivity analysis by baseline carried forward method confirmed all independent predictors of 6-month weight loss and reduced the model fit by only 11%. The high success group lost weight faster and maintained weight loss more efficiently than the other groups (*P*<.001). Early weight loss was associated with weight maintenance after 1 year and 2 years (both *P*<.001). Weight dynamics did not differ between men and women over 6 months when adjusted for baseline and usage parameters (*P*=.91). The percentage of male long-term users was unusually high (42.2%).

**Conclusions:**

Our results suggest that early weight loss and close program adherence (ie, 5 dietary protocols per week and weekly entering of current weight), especially in the early phase of program usage, can improve weight loss outcome.

## Introduction

In 2008, more than 50% of European men and women were overweight [1]. Obesity was present in 21% of women and 22% of men aged 20 years or older [2]. Between 1980 and 2008, the prevalence of obesity nearly doubled worldwide [1]. During the same period, Internet availability and usage also increased significantly. In the European Union, the percentage of citizens aged between 16 and 74 years with Internet access at home increased from 33% in 2004 to 67% in 2011 [3]. Furthermore, 38% of Europeans searched the Web for health-related information in 2011 [4]. This suggests that weight loss programs delivered via the Internet have the potential to reach and be accepted by large numbers of European citizens. Commercial weight loss platforms are steadily increasing in number and warrant special attention.

Web-based weight loss programs provide a health intervention that is flexible, timesaving, and cost-effective [5]. For weight loss interventions with increased intensity (eg, treatment by a doctor or other professionals), overweight and obese individuals reported an increasing number of obstacles (eg, lack of money or time, disgrace) [6]. Web-based programs overcome traditional access barriers of face-to-face counseling (eg, by protecting user anonymity [7] or by reducing travel times [8]), thus appealing to broad levels of the population [9]. Several Web-based weight loss interventions have been shown to be efficient in supporting weight loss [10-14]. Two systematic reviews with meta-analysis on Web-based weight loss interventions found that Internet-based programs have the potential to achieve weight loss and can result in weight loss outcomes comparable to other weight loss interventions [15,16]. Online tools that visualize goal progress or feedback, such as a body mass index (BMI) calculator or progress graphs, were found to be especially supportive in weight reduction [17].

Since 2005, a commercial online weight loss program that incorporates both feedback and visualizing tools has been available in German-speaking countries (KiloCoach). A previous study showed that KiloCoach users who continuously entered dietary protocols for at least 60 days (n=946) lost 4.1% (SD 5.5) of their baseline weight [18]. Program users who entered protocols for 1 year (n=104) lost 6.4% (SD 7.3) of their baseline body weight [18].

The primary objective of the present work was not to evaluate the overall weight loss efficacy of this program, but to investigate the weight loss dynamics of KiloCoach users who used the program for at least 6 months and to associate final weight loss with the use of different program tools. Based on our results, we aimed at drafting user recommendations on how to use the program more effectively in future.

## Methods

### Program Description

KiloCoach is available on the Web [19]. The key concept of this commercial program is to induce lifestyle changes that lead to weight loss. Users are encouraged to adapt healthier eating and activity habits by means of self-monitoring combined with tailored feedback and information about health and nutrition.

**Figure 1:**
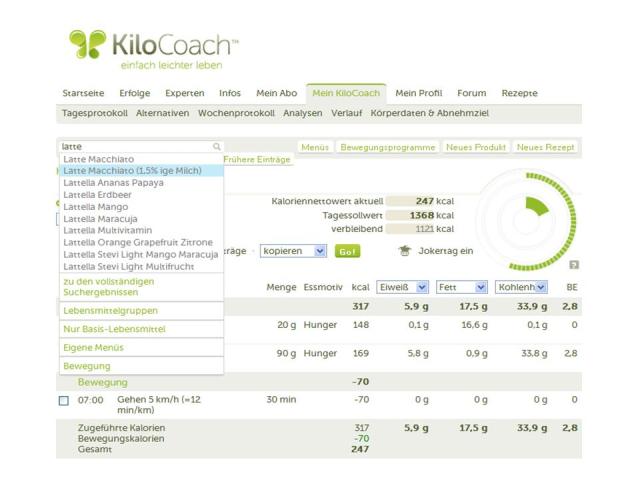
Screenshot of a dietary protocol in KiloCoach (see Multimedia Appendix 1 for explanation).

Self-monitoring includes optional logbook and weight entries in addition to dietary protocols (Multimedia Appendix 1). The most important program tool is the dietary protocol, which is the electronic version of the common written protocols for recording food intake (Figure 1). It provides the electronic facility to quickly record all food items and drinks from a database of approximately 40,000 items. Additionally, physical activity can be recorded daily. Based on the dietary protocol, energy intake and expenditure are calculated, analyzed, and visualized in real time to provide immediate feedback to the user. The logbook is a private blog that offers the opportunity to document special situations or additional anthropometric measurements. Thus, logbook entries are defined as small personal notes entered by the user. A weight entry is a weight recorded by the user. To assist with weight reduction, KiloCoach calculates an upper threshold for daily energy intake (kcal) based on a user’s body data and individual weight loss goal, considering that weight loss should not exceed 1 kg per week. Further supportive features are analyzing tools that analyze diet composition or identify food groups that mainly contribute to energy intake, for example (Multimedia Appendix 1). Finally, users can actively participate in the weight loss community’s online forums and contact nutrition, sports, coaching, as well as medical experts, if required.

Because KiloCoach is based on a healthy diet, encourages participants to increase physical activity, and anticipates a weight loss speed of 0.5 to 1.0 kg per week, it fulfills the internationally accepted criteria for recommendable weight loss programs [20].

Datasets from KiloCoach users who became members for at least 2 months between January 1, 2008 and December 31, 2010, and who did not report confounding factors to weight loss (diabetes mellitus, hypothyroidism, limited motility, pregnancy, or lactation) were eligible for the present analysis.

Additional inclusion criteria were at least 1 dietary protocol available during program use, no program interruption longer than 3 months, age ≥ 18 years, and BMI between 27 to 39.9 kg/m^2^ [10,21]. The BMI cut-off points were chosen to allow the direct comparison of this retrospective analysis with the results of a prospective controlled trial that is currently being carried out in our center (clinicaltrials.gov registration: NCT01634204) and with other studies already published.

All 1123 datasets fulfilling the predefined criteria were extracted by KiloCoach and sent unmodified to the Charité - Universitätsmedizin Berlin. The datasets of users who were still active after 6 months were selected by the Charité - Universitätsmedizin Berlin. This selection was done first because various guidelines on the treatment of overweight and obesity recommend a weight loss duration of 6 months [20,22]. Moreover, a duration of 6 months is used in many international weight loss trials, which allows for comparisons.

**Figure 2:**
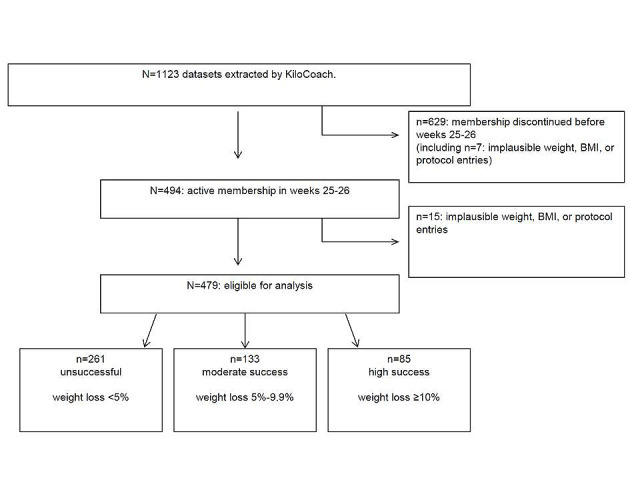
Dataset flow through the screening process to success group allocation.

The resulting sample eligible for analysis, referred to as study sample, contained 479 datasets (Figure 2). An observation period from January 1, 2008 to December 31, 2011 was chosen to allow a usage period of at least 12 months for every user.

Each dataset contained self-reported personal data, such as age, sex, height, and body weight. The BMI was calculated from self-reported weight and height. Additional data were duration of membership, number of purchased membership days, and indicators for program usage and compliance expressed as frequencies of dietary protocols, weight entries, logbook entries, and meals per day. As expected for a self-guided program, not every user entered his or her weight at the same time or used the program following the same pattern. Thus, we averaged weight and BMI over periods of 2 weeks for the first 6 months (weeks 1-2, weeks 3-4,..., weeks 25-26). Frequencies of dietary protocols, weight entries, and logbook entries were expressed in absolute numbers over the same time periods.

Users were divided into 3 weight loss success groups, referred to as unsuccessful, moderate success, and high success. These groups were based on an achieved percentage weight loss of <5%, 5%-9.9%, and ≥10% of initial weight, respectively, after 6 months of program usage. This classification was chosen because weight reduction lower than 5% is considered insignificant [22], between 5% and 10% is moderate [23], and above 10% is high [24].

Data entries after the 6-month period (after weeks 25-26) were referred to as follow-up because the weight loss phase changes into weight maintenance after 6 months [20]. For follow-up data, intervals were created for 1, 1.5, and 2 years (weeks 47-57, weeks 72-84, and weeks 93-114, respectively).

### Statistical Analysis

#### Overview

Statistical analysis was carried out with SPSS version 19 (IBM Corp, Armonk, NY, USA), and *P* values <.05 were considered statistically significant. Descriptive results are given as means and standard deviations (SD) if not indicated otherwise. Missing system values for the numbers of dietary protocols and logbook entries originated from nonuse in the respective time periods and were consequentially replaced by 0 numbers resulting in exact and complete information on program usage.

In the study sample, 39.31% of possible weight entries (2448/6227) were missing because of the self-guided character of the program. For example, users were not aware to actively re-enter weight during periods of weight stabilization, which often exceeded 2 weeks during active weight loss attempts. Overall, 1187 of 6227 weight entries (19.06%) were unavailable between 2 active weight entries. Only 1261 of 6227 (20.25%) were missing after the last weight entry, and 1013 (80.33%) of these belonged to the unsuccessful group, when the last observation carried forward method (LOCF) was used to complement missing values. Consequentially, only 248 weight data were missing after the last weight entry in the moderate success and high success groups (8.75% of moderate and high or 3.98% of total sample). There is no consensus on how to deal with missing values [25]. From a clinical standpoint, LOCF provided the best estimate to complement missing values of self-reported weight in this scenario. Consequentially, we chose LOCF imputation for primary analysis.

Nevertheless, we additionally performed a sensitivity analysis by using the baseline carried forward (BCF) method, a more conservative estimate for completing missing values. In our sample, which is characterized by a high number of in-between missing weight entries, BCF leads to clinically implausible weight in users with significant weight loss. It also results in a worst-case scenario for success group allocation, because all users who did not enter weight in weeks 25-26 were allocated to the unsuccessful group even when a significant weight loss was confirmed by active weight entries 2 weeks before (see subsequent sensitivity analyses also). This negative scenario was considered adequate to test the robustness of LOCF results.

Missing weight data in the follow-up period (after weeks 25-26) were not complemented by LOCF or any other imputation.

#### Linear Analysis by General Linear Model Repeated Measures and Multivariate Regression Analysis

Changes from baseline in outcome measures were analyzed with a general linear model (GLM) for repeated measures. In a 14-level model time (duration of platform usage), group (success group), and sex were tested and adjusted for baseline values and usage markers (age, BMI, number of protocols, and number of weight entries as covariates). The 13 degrees of freedom (df) contrast describing the difference in trajectories over time among success groups was taken as a primary indication of different weight dynamics dependent on final weight reduction. The 13-df contrast describing the difference in trajectories over time between men and women was taken as a primary indication of different weight dynamics dependent on sex.

We applied multivariate linear regression analyses controlled for collinearity (variance inflation factor <3), autocorrelation (Durbin-Watson statistic=1.18), and outliers (standardized residues <3.2) to evaluate the impact of early weight loss, baseline characteristics, and user behavior on total weight loss at 6 months in the primary analyses and the sensitivity analyses. In the primary analyses, case-wise diagnoses identified 2 participants with extreme weight loss of 18% to 20% (no: 617 and 923), who significantly influenced the results (*z* scores > 3.1). We kept both participants in the model. Omitting their data would have increased the adjusted multivariate coefficient of determination (adjusted *R* ^*2*^) by 2% without changing the main results of the analyses. Thus, all participants were included in the analyses (N=479).

#### Post Hoc Analysis

The post hoc analysis of differences among all groups was done with the Kruskal-Wallis test for continuous and discrete variables and the chi-square test (χ^2^) for binary variables. Two group comparisons were performed using the Mann-Whitney *U* test; Spearman rank correlation (ρ) was used for bivariate analyses. All post hoc analyses were exploratory; therefore, no Bonferroni adjustment was applied [26]. In some figures, box-whisker plots were used displaying the 25th, 50th, and 75th percentiles in the boxes and the minimum and maximum as whiskers, except for extreme values.

#### Sensitivity Analyses

To test the robustness of the LOCF imputation, we conducted a sensitivity analysis. First, we used the BCF method to complement all missing weight data within the first 6 months of program usage in long-term users (N=479).

Second, we evaluated separately the subgroup of users who coincidentally entered their body weight at our endpoint for weight loss, weeks 25-26 (N=214). In this group, final weight was self-reported by all users and only in-between missing weight entries had to be completed by LOCF. This sample provides the most probable reproduction of weight dynamics and success group allocation, albeit at the cost of group size and representability.

## Results

### Kilocoach Users With and Without Weight Entry After Six Months

KiloCoach users who actively used the platform for at least 6 months were referred to as long-term users and were chosen as the study sample (N=479). The study sample accounted for 42.7% of the total KiloCoach population.

Baseline weight, height, and BMI were statistically different between long-term and short-term users, but the numeric difference was insignificant, as shown in Table 1. The study sample had a higher proportion of men and, as expected, significantly more purchased membership days compared to short-term users.

**Table 1:** General characteristics of long-term users (≥6 months activity), short-term users (<6 months activity), and all users of KiloCoach.

General characteristics	Total population N=1123	Long-term users n=479	Short-term users n=644	*P* ^a^
Sex (male), n (%)	435 (38.7)	202 (42.2)	233 (36.2)	<.001
Age (years), mean (SD)	44.2 (11.8)	44.0 (11.7)	43.8 (11.8)	.20
Initial weight (kg), mean (SD)	92.5 (13.8)	94.4 (14.4)	92.0 (14.3)	<.001
Height (cm), mean (SD)	172 (8.7)	172 (8.8)	171 (8.6)	.04
BMI (kg/m^2^), mean (SD)	31.3 (3.1)	31.7 (3.2)	31.0 (3.0)	<.001
Purchased membership days, mean (SD)	275 (236)	413 (264)	172 (143)	<.001

^a^Long-term vs short-term users; Mann-Whitney *U* test used except for sex (chi-square test used).

### Primary Analysis of the Dynamics and Predictors of Weight Loss

#### General Characteristics of the Success Groups

Table 2 shows the baseline characteristics of the KiloCoach study sample. Of the entire sample, 54.5% (261/479) were unsuccessful (lost <5% initial body weight), 27.8% (133/479) were moderately successful (lost 5% to 9.9% initial body weight), and 17.8% (85/479) were highly successful (lost ≥10% initial body weight). Six-month weight reduction significantly differed among the success groups and averaged 5.3% (SD 5.6) in the total group. Users were aged between 18 and 74 years. Sex distribution, age, baseline body weight, height, and baseline BMI did not differ significantly among the success subgroups, although the number of purchased membership days differed significantly among the groups (Table 2).

**Table 2:** General characteristics of the study sample according to the percentage weight loss achieved after 6 months: unsuccessful (lost <5% of initial body weight), moderate success (lost 5%-9.9% of initial body weight), and high success (lost ≥10% initial body weight).

General characteristics	All (N=479)	Unsuccessful (n=261)	Moderate success (n=133)	High success (n=85)	*P* ^*a*^
Sex (male), n (%)	202 (42.2)	111 (42.5)	49 (36.8)	42 (49.4)	.18
Age (years), mean (SD)	44.0 (11.7)	43.6 (11.6)	46.2 (11.4)	46.2 (12.2)	.08
Initial weight (kg), mean (SD)	94.4 (14.4)	95.2 (14.9)	92.9 (4.9)	94.4 (14.1)	.26
Height (cm), mean (SD)	172 (8.8)	173 (9.3)	172 (8.3)	172 (8.0)	.77
BMI (kg/m^2^), mean (SD)	31.7 (3.2)	31.9 (3.4)	31.3 (3.0)	31.6 (3.1)	.28
Purchased membership days, mean (SD)	413 (264)	374 (245)	420 (247)	521 (312)	<.001
6-month weight loss (%), mean (SD)	5.3 (5.6)	1.2 (2.4)	7.4 (1.5)	14.2 (3.8)	<.001

^a^Kruskal-Wallis 1-way ANOVA over the 3 subgroups used except for sex (chi-square test used).

### The Dynamics of Weight Loss

The weight loss dynamics showed significant differences among the 3 groups over the weight loss period of 6 months (GLM repeated measures, 13 df contrast, *P*<.001).

**Figure 3:**
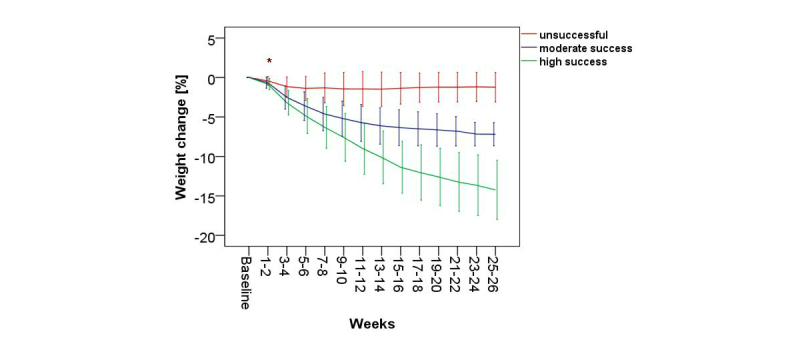
Percentage weight loss over 6 months for the unsuccessful (<5% weight loss), moderate success (5%-9.9% weight loss), and high success (≥10% weight loss) groups using KiloCoach. *= start of significant difference among the 3 subgroups (Kruskal-Wallis 1-way ANOVA, *P*<.001).

The post hoc analysis showed that, despite similar initial weight and BMI, weight reduction after 2 weeks of KiloCoach usage already differed significantly among the 3 groups, with a mean of 0.4% (SD 0.6) in the unsuccessful group, 0.7% (SD 0.7) in the moderate success group, and 0.9% (SD 0.7) in the high success group (*P*<.001) (Figure 3). For all the following time points, weight loss differed significantly among the 3 success groups (*P*<.001). Furthermore, compared to the moderate success group, the high success group achieved significantly more weight reduction from weeks 3-4 onward (mean 3.2%, SD 1.5 vs mean 2.5%, SD 1.5, *P*<.001). The high success group lost weight faster than the other groups. Although both the moderate and the high success groups reached their maximum percentage weight loss, weight loss was significantly more in the high success group (*P*<.001) after 6 months. This finding was in contrast to the unsuccessful group who lost weight only until weeks 11-12. Achieved weight loss remained stable in the unsuccessful group until weeks 15-16, but was followed by weight regain by weeks 25-26. Multimedia Appendix 2 provides a detailed description of the course of weight loss in all groups.

### Bivariate Correlations to Identify Indicators for Successful Weight Loss

#### Baseline Characteristics

Baseline body weight and BMI (rho=–0.027, *P*=.55; rho=–0.034, *P*=.46, respectively) and user’s sex (rho=–0.053, *P*=.25) did not correlate with percentage weight reduction after 6 months in the unadjusted bivariate analysis; however, a positive correlation was observed for the user’s age (rho=0.138, *P*=.002).

#### Early Weight Loss

**Figure 4:**
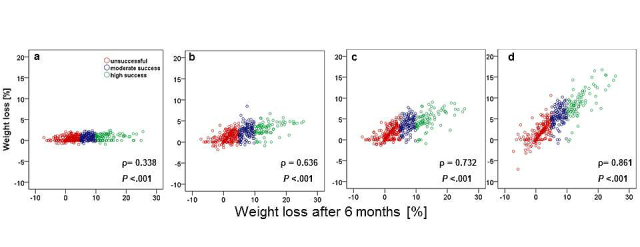
Correlation (Spearman rho, ρ) between early weight loss and weight loss outcome after 6 months during weeks 1-2 (a), weeks 3-4 (b), weeks 5-6 (c), and after 3 months (d) for the unsuccessful (<5% weight loss), moderate success (5%-9.9% weight loss), and high success (≥10% weight loss) groups using KiloCoach.

We already observed a significant positive correlation (*P*<.001) with final weight loss in the first 2 weeks of program usage (Figure 4). The correlation became steadily stronger during the following 4 weeks, reaching an association comparable to the 3-month outcome by weeks 5-6 (Figure 4).

### Program Usage

**Figure 5:**
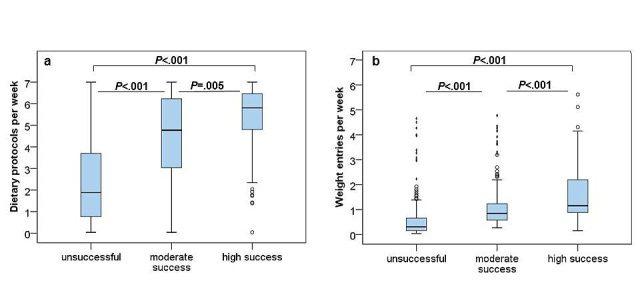
Use of program tools in the success groups during the first 6 months of program usage. Weekly number of dietary protocols (a) and number of weight entries (b) differed significantly among the unsuccessful (<5% weight loss), moderate success (5%-9.9% weight loss), and high success (≥10% weight loss) groups.

The weekly number of dietary protocols, weight entries, and logbook entries differed significantly among success groups over 6 months (all *P*<.001). These weekly numbers were significantly lower for the unsuccessful group compared to the moderate success group (*P*<.001; Figure 5), and the moderate success group had significantly less dietary protocols (*P*=.005), weight entries (*P*<.001), and logbook entries (*P*=.001) per week than the high success group. For numeric results, see Multimedia Appendix 2. After 6 months, the 3 program tools with the strongest correlations with percentage weight loss after 6 months were weekly numbers of dietary protocols (rho=0.589), weight entries (rho=0.631), and logbook entries (rho=0.599, all *P*<.001).

Reported energy intake was lowest in the unsuccessful group and highest in the high success group (unsuccessful: mean 1705 kcal/d, SD 607; moderate success: mean 1984 kcal/d, SD 675; high success: mean 2156 kcal/d, SD 740, *P*<.001 among groups) and correlated positively with weight loss after 6 months (rho=0.22, *P*<.001). In-line with this finding, the number of daily meals increased with increasing success (rho=0.247, *P*<.001). Both variables are interpreted as indicators for the accuracy of the dietary protocols rather than as objective measures of dietary intake.

The use of all program features decreased with increasing usage period in all success groups. For example, 70.1% (SD 21.2) of all dietary protocols present after 6 months of KiloCoach use were written within the first 3 months.

### Multivariate Analysis

We performed a multivariate analysis to investigate the impact of early weight loss and program usage adjusted for possible confounding factors, such as sex, age, and baseline BMI. Percentage weight loss as a continuous variable after 6 months was chosen as the dependent variable. The total number of logbook entries showed strong collinearity to the total number of weight entries (variance inflation factor=16.1) and had to be removed from the model. The model is summarized in Table 3. The *F* test disclosed significant associations (*P*<.001) with the adjusted *R* ^*2*^, indicating that 52.4% of the variance in weight loss was explained by the model. Early weight loss by weeks 3-4, the total number of protocols, and the total number of weight entries qualified as independent predictors of 6-month weight reduction. The model summary was *R*=0.728, adjusted *R* ^*2*^ =0.524, *F*_6,472_=88.6, *P*<.001. Replacing weight loss by weeks 3-4 with weight loss during the first 2 weeks as dependent variable resulted in a considerable deterioration of the model fit by reducing the adjusted *R* ^*2*^ to 39.7%.

### Sensitivity Analyses

Sensitivity analyses were carried out using BCF imputation and subgroup evaluation.

#### Baseline Carried Forward Imputation

Missing weight data were imputed using baseline weight. Because success groups were based on percentage weight loss after 6 months, group sizes changed using this model. The unsuccessful group increased to 344 users, whereas the moderate and high success groups decreased to 71 and 64 users, respectively. The average weight loss after 6 months was 3.3% (SD 5.4) in the total group (Multimedia Appendix 3).

**Figure 6:**
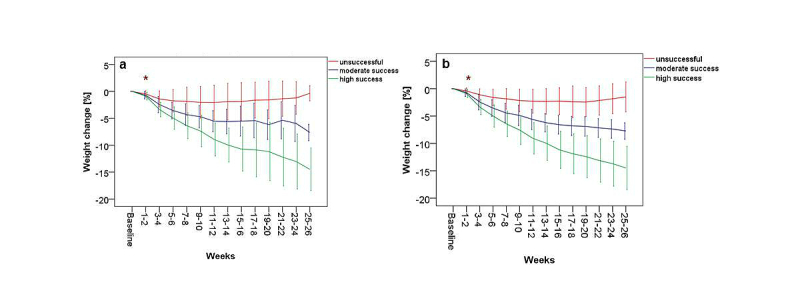
Sensitivity analysis. Development of percentage weight loss in the unsuccessful (<5% weight loss), moderate success (5%-9.9% weight loss), and high success (≥10% weight loss) groups when missing weight data in the study sample were imputed using the baseline carried forward (BCF) method (a) and when only users with active weight entry in weeks 25-26 were included and missing data were imputed using last observation carried forward (LOCF) method (b). * = start of significant difference among the 3 subgroups (Kruskal-Wallis 1-way ANOVA, P<.001).

Results of the primary analysis were confirmed for the weight loss dynamics in the GLM repeated measures (13 df contrast, *P*<.001) (Figure 6) and in the post hoc evaluation because weight loss among the 3 groups differed already significantly in weeks 1-2 (*P*<.001) and weight loss was higher in high compared to moderate success group from weeks 3-4 onward (*P*<.001).

Multivariate analysis confirmed the results of the primary analysis. Early weight loss by weeks 3-4, total number of protocols, and total number of weight entries qualified as independent predictors of 6-month weight reduction (all *P*<.001) in a model summary (*R*=0.644, adjusted *R* ^*2*^=0.412, *F*_6,472_=131.9, *P*<.001).

**Table 3:** Multiple regression analyses including regression coefficients (b) and standardized regression coefficient (ß-weight) for predicting percentage weight reduction after 6 months of program usage.

Multiple regression	b	*t* test	
		ß-weight	*P*
Weight loss week 3-4 (%)	1.579	0.457	<.001
Sex (female= 0, male =1)^a^	–0.015	–0.001	.97
Age (years)	0.008	0.018	.59
Baseline BMI (kg/m^2^)	0.060	0.035	.27
Total protocols (n)	0.027	0.286	<.001
Total weight entries (n)	0.040	0.188	.001

^a^Dummy-coded term.

#### Subgroup Evaluation of Users With Six-Month Weight Entry

Only users who entered a weight in weeks 25-26 were included in this analysis (n=214). This subgroup was chosen because of its superior data quality. No after last entry weight data had to be imputed in this sample, rendering it most concise for final weight loss and allocation to success groups. The sizes of the success groups changed: unsuccessful (n=79), moderate success (n=71), and high success (n=64). The subgroup analysis resulted in a 6-month weight loss of 7.5% (SD 6.0) (Multimedia Appendix 3).

Results of the primary analysis were confirmed for the weight loss dynamics in the GLM repeated measures model (13 df contrast, *P*<.001) (Figure 6) and in the post hoc analysis. Again, weight loss differed significantly between the 3 success groups after 2 weeks of program usage (*P*<.001).

Multivariate analysis confirmed the results of the primary analysis. Early weight loss by weeks 3-4 (*P*<.001), total number of protocols (*P*<.001), and total number of weight entries (*P*=.002) qualified as independent predictors of 6-month weight reduction. The model summary was *R*=0.591, adjusted *R* ^*2*^=0.330, *F*_6,211_=18.8, *P*<.001.

### Gender Differences

The weight dynamics were similar between men and women in the trajectory over time during the weight loss period of 6 months when adjusted for age, BMI, number of protocols, and number of weight entries (GLM repeated measures, 13 df contrast, *P*=.91).

In the post hoc bivariate analysis, age, baseline BMI, and purchased membership were comparable between men and women (Table 4). Percentage 6-month weight loss and assignment to success groups were not statistically different between the sexes (Table 4).

**Figure 7:**
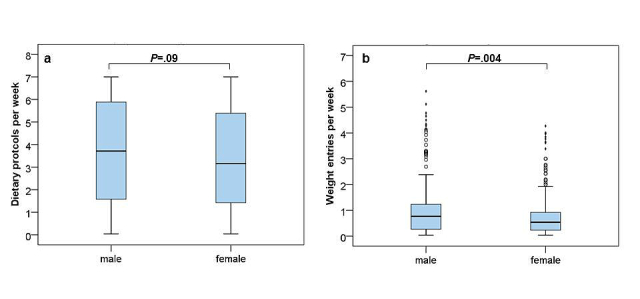
Gender-specific use of program tools in the first 6 months of program participation. Dietary protocols (a) were used more frequently by men, who also entered significantly more body weights per week (b) than women.

Over 6 months, males entered significantly more weight entries (*P*=.004) and logbook entries per week than females (*P*=.004). Additionally, males tended to write more dietary protocols than females (*P*=.09) although this did not reach statistical significance (Figure 7).

**Table 4:** Gender differences of the study sample.

Gender difference		Male	Female	*P* ^a^
n		202	277	.001
Age (years), mean (SD)		44.4 (11.2)	45.1 (12.0)	.44
Initial weight (kg), mean (SD)		102.0 (13.3)	88.7 (12.3)	< .001
Baseline BMI (kg/m^2^), mean (SD)		31.7 (3.3)	31.6 (3.2)	.99
Purchased membership days, mean (SD)		441 (280)	393 (250)	.13
**Group distribution, n (%)**				.18
	Unsuccessful	111 (55.0)	150 (54.2)	
	Moderate success	49 (24.3)	84 (30.3)	
	High success	42 (20.8)	43 (15.5)	
6-month weight loss (%), mean (SD)		5.7 (5.7)	4.9 (5.4)	.25

^a^Mann-Whitney *U* test used except for proportions in success groups (chi-square test used).

### Weight Maintenance

**Figure 8:**
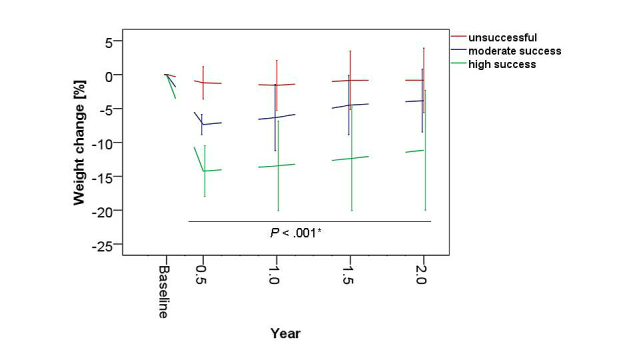
Development of percentage weight loss in the success groups compared to baseline. Weight maintenance during the follow-up period (1, 1.5, and 2 years after the first dietary protocol) of the unsuccessful (<5% weight loss), moderate success (5%-9.9% weight loss), and high success (≥10% weight loss) groups of the study sample. * = significant difference among the 3 subgroups (Kruskal-Wallis 1-way ANOVA).

At the end of the follow-up period (ie, the time between weeks 25-26 and 2 years after the first dietary protocol), the unsuccessful group maintained a slight body weight reduction of 0.8% (SD 4.7) compared to baseline weight (Figure 8). Although some weight regain was observed in the moderate success group, the weight loss of 3.9% (SD 4.6) differed significantly from that of the unsuccessful group (*P*=.006) after 2 years. Although the high success group also regained some weight after 2 years compared to their weight at 6 months, this group maintained a clinically significant weight loss of 11.2% (SD 8.9) and differed significantly from the unsuccessful (*P*<.001) and moderate success (*P*=.001) groups. See Multimedia Appendix 2 for a detailed description of the weight maintenance period.

There were still significant positive correlations between early weight loss in weeks 1-2 and weight maintenance up to 2 years (Table 5). Also during follow-up, no significant gender-related maintenance pattern was observed.

**Table 5:** Spearman correlations (ρ) between early weight loss and weight maintenance in the study sample.

% Weight loss (long term)	% Weight loss (early)					
	Weeks 1-2		Weeks 3-4		After 3 months	
	ρ	*P*	ρ	*P*	ρ	*P*
After 1 year	0.249	<.001	0.402	<.001	0.604	<.001
After 1.5 years	0.237	.007	0.396	<.001	0.547	<.001
After 2 years	0.278	.008	0.447	<.001	0.553	<.001

## Discussion

### Principal Findings

The KiloCoach online program aims at supporting self-guided body weight reduction and provides information, tools for self-monitoring and analysis of eating habits, and social support. Our aim was to identify predictors of weight loss effectiveness in long-term users of KiloCoach in a real world setting. *Long term* was defined as program adherence of at least 6 months. This criterion was fulfilled by 44% of KiloCoach users who represent a relevant proportion of the total KiloCoach population.

We showed that, despite comparable baseline characteristics, weight loss dynamics and weight maintenance differed significantly among the 3 success groups of our study sample. Multivariate analysis showed that early weight loss (weeks 3-4), number of dietary protocols, and number of weight entries to be independent predictors for final weight loss after 6 months in the primary analysis confirmed by 2 sensitivity analyses. Moderate and high successors used program tools more frequently than unsuccessful users. The total population and study sample consisted of an unexpectedly high percentage of male users, who demonstrated weight reduction comparable to females and even higher intensity of program usage.

### Early Weight Loss

So far, 3 trials have reported that early weight loss is positively related to final weight loss [27-29]. All trials evaluated in-person weight loss programs. In the DiOGenes study, early weight loss after 1 and 3 weeks of dieting predicted weight loss after 8 weeks on a low-calorie diet (800 kcal/d) [29]. Fabricatore et al [27] found that early weight loss after 3 weeks of treatment predicted successful weight loss after 1 year. Elfhag et al [28] reported that weight loss after 5 weeks of treatment best forecasted weight loss after 8 to 10 months of group sessions.

To our knowledge, this is the first time that the effects of early weight loss on later weight loss and weight maintenance have been evaluated for a commercial online weight loss program. We found weight loss in the third and fourth week of platform use was highly predictive for the 6-month outcome and was also significantly associated with weight maintenance up to 2 years. Interestingly and against intuition, early weight loss does not seem to be associated with pretreatment motivation [28], a psychological factor that could not be evaluated in the present study. However, self-motivation quickly fluctuates in relation to lapses and relapses [30]. Users with significant early weight loss were likely to have reinforced their motivation during program use and thereby increased self-efficacy to lose weight. Early weight loss might be a modifiable factor that could be influenced by educational advice supplied by the platform provider.

### Program Usage

By means of weight tracking, dietary protocols, and analyzing tools, KiloCoach users are encouraged to self-monitor and change their lifestyle. Previous studies showed that self-monitoring behavior of weight, diet, and activity are cardinal behaviors of successful weight controllers [30-32]. Our results also depict that usage intensity of KiloCoach was associated with higher weight loss. Especially the features of self-monitoring weight and diet were predictive for 6-month weight loss, even when adjusted for early weight loss. These results are in-line with the finding of Krukowski et al [33] that overall online self-monitoring is associated with weight loss outcome after 6 months. They further described that the achievement of weight loss greater than 5% was more likely in users who consistently self-monitored in the early usage phase (ie, within the first 4 weeks).

Food and exercise diaries were previously identified as the most effective program features for weight loss in retrospective studies [12,14] and in 1 prospective randomized trial [34]. Adherence to the old method of paper dietary protocols is a well-known problem; it is a common experience that documenting more than 7 days in succession leads to inadequate results because of decreased interest and boredom of the individuals [35]. Thus, we were surprised to find a high mean number of online dietary protocols over 6 months ranging from mean 2.4 (SD 2.0) per week in the unsuccessful group to mean 5.3 (SD 1.7) per week in the high success group. The mean total number of dietary protocols was 63 and 137, respectively, meaning that users generated dietary protocols on 35% to 75% of their participation days. Computer-based technology seems to facilitate self-monitoring of diet, and further technical advancements of Web-based programs are expected to continuously improve self-monitoring adherence [36,37].

Both energy intake and number of meals were significantly higher in the high success group than in the unsuccessful group, which appears idiosyncratic with regard to successful weight loss. These 2 factors are most likely indicators for the average quality of dietary protocols, which suggests that dietary protocols were filled in more thoroughly by the high success group.

### Male Users

It was surprising to observe a percentage of 39% male users in the total KiloCoach population and of 42% in the study sample. Comparing this finding to other non-Web-based weight loss interventions, this proportion is very high. For example, only 3.6% of all users of a special offer to access Weight Watchers were men [38]. In a workplace-based weight loss program, only 6.6% of the enrolled participants [39] and 7.4% of the completers [40] were men. Considering the lower barriers for participation in online weight loss programs, such as anonymity, flexible integration into everyday life, and the self-guided procedures, one could assume that men are particularly attracted by such an intervention. Still, the proportion of male users in KiloCoach is high, even compared with other Web-based weight loss programs. Other programs reported male participation ranging from 14% [12,41] to 26% [14,15].

Currently, we can only speculate about the reasons why KiloCoach is more attractive to men. According to the program owner, the website and the user interface of the program are designed to appeal to men and women. However, men and women seem to prefer different program features. For example, men seem to prefer calculative tools, especially for weight forecasts, whereas women seem to be more interested in nutritional information, including the calculation of one’s own recipes, and peer support (personal communication).

### Effectiveness

We reported an average weight loss of 5.3% (SD 5.6) of baseline weight after 6 months of KiloCoach usage in our primary analysis. This finding confirms previous results using the same platform (weight loss of 4.4%, SD 5.1 and 6.4%, SD 7.3 in users who followed the program for at least 60 days or still entered dietary protocols after 1 year) [18]. The program shows a satisfying overall result compared to structured, in-person weight loss programs that usually average between 5% and 10% weight loss over 6 months [42,43]. The proportion of our long-term users who lost weight successfully (ie, ≥5% of baseline weight) was 46%. For comparison, Krukowski et al [33] showed that 53% of participants in their online study arm condition achieved a weight loss of 5% or more after 6 months. In another study, a smaller proportion (36%) of study completers achieved this weight loss [44].

A recent Cochrane review showed that structured in-person treatment resulted in a mean weight loss that was only 2.1 kg higher than that achieved by Web-based intervention [15]. When compared with minimal personal interventions (eg, information, standard care), Web-based programs lead to an even higher weight loss of 1.5 kg after 6 months [15]. Thus, Web-based weight loss intervention programs are a feasible and efficient compromise between high and minimal resource interventional programs.

With regard to weight maintenance, the high success group maintained a clinically significant weight loss after 2 years. This finding also points toward a possible advantage over other successful weight loss programs because individuals often fail to maintain their weight loss over longer periods of time [24].

### Strengths and Limitations

A strength of our work is that, for the first time, a weight loss platform for the German-speaking countries Germany, Austria, and Switzerland was investigated concerning weight loss dynamics and usage of program tools. Another strength is that all long-term users were evaluated. Thus, the results are representative for this group. Furthermore, we showed with this study that detailed long-term electronic documentation of nutrition and exercise seems to be possible.

The retrospective design might be seen as a disadvantage, but it presented an immediate opportunity to investigate existing data of the KiloCoach database, representing field data from the real world in a population in which scientific evaluation was not prospectively intended. This approach can be advantageous because nutritional behavior is responsive to observation, which might bias the results.

Critics might further note that all information about the users is self-reported. Body weight might be especially underreported, but Harvey-Berino et al [45] found that self-reported weight is highly accurate and weight loss conveyed by self-reporting is comparable to de facto weight loss. Users included in this study had to buy a membership for program usage and had to lose weight on their own; they would not have benefited from program usage if the reported data did not agree with reality.

One limitation of our study is the absence of information about motivation at the beginning of program use or demographic characteristics other than age and sex (eg, ethnicity, marital status, education) that might have influenced weight loss. Problems in identifying predictors have been summarized recently [30].

Meanwhile, a controlled prospective trial has started to further increase the knowledge about efficacy and weight dynamics in KiloCoach users.

### Conclusion

KiloCoach is an effective self-help tool to reduce weight that attracts more men than other programs and enables long-term monitoring of dietary intake and physical activity. To achieve the best possible weight loss result, users should closely adhere to the program for frequent and thorough usage of self-monitoring tools. To be more specific, writing dietary protocols 5 days per week and entering body weight on 1 day per week is recommended according to our results. Close program adherence seems to be especially important during the first period of program usage, with the aim to induce early weight loss and improve chances for further success.

## Multimedia Appendix 1

KiloCoach dietary protocol and analyzing tools.

## Multimedia Appendix 2

Detailed description of weight loss and weight maintenance period and of program usage intensity.

## Multimedia Appendix 3

Sensitivity analyses - weight change results.

## Abbreviations

BCF: baseline carried forward
BMI: body mass index
GLM: general linear model
LOCF: last observation carried forward
