# Supplementary material for: Determinants of Successful Weight Loss After Using a Commercial Web-Based Weight Reduction Program for Six Months: Cohort Study
Source: J Med Internet Res. 2013 Oct 14;15(10):e219. doi: 10.2196/jmir.2648 (PMC3806515; doi:10.2196/jmir.2648)
Supplement: Supplementary file 1 [file jmir_v15i10e219_app1.ppt]

## Slide 1
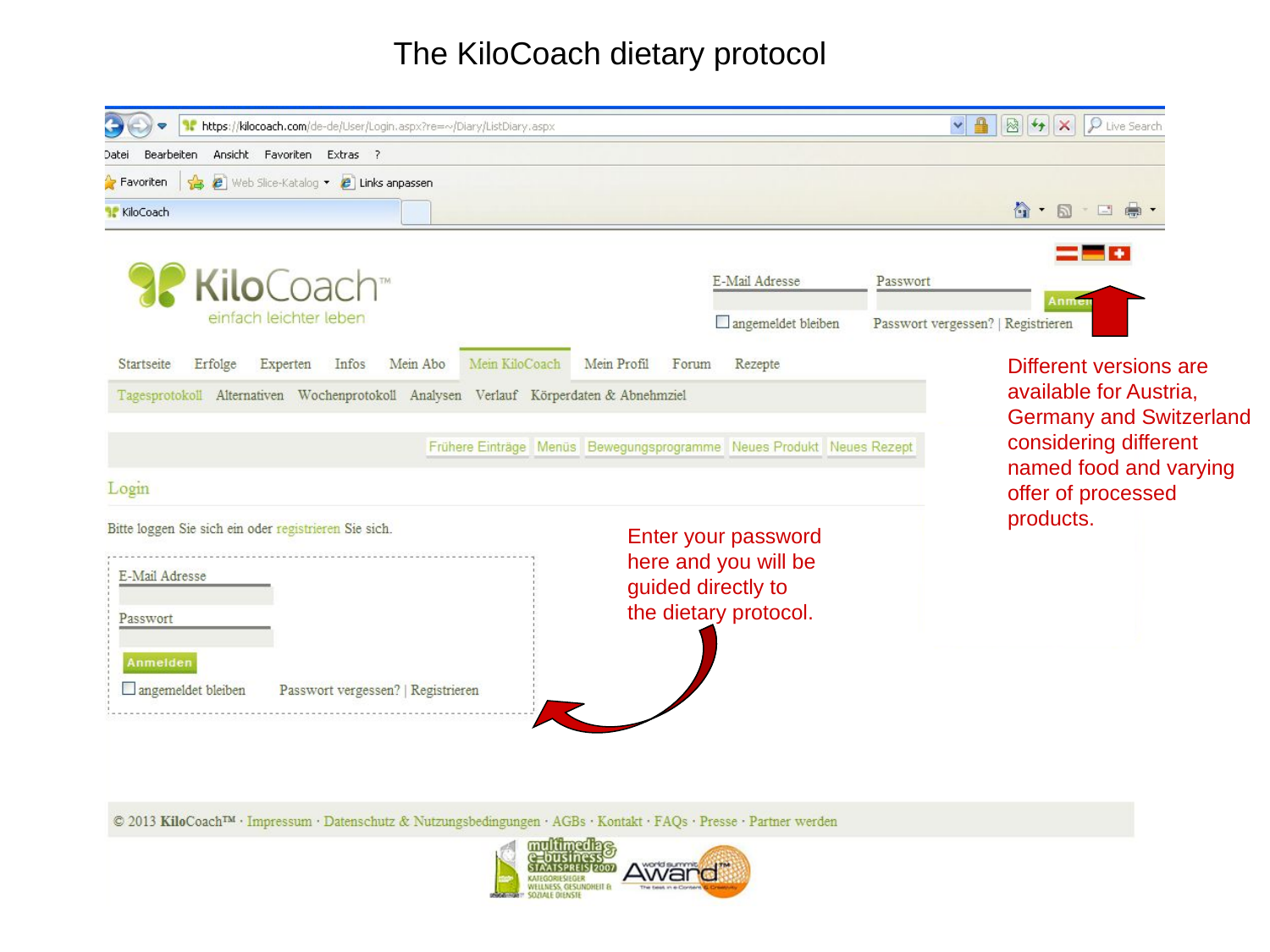

The KiloCoach dietary protocol
Different versions are available for Austria, Germany and Switzerland considering different named food and varying offer of processed products.
Enter your password here and you will be guided directly to the dietary protocol.

## Slide 2
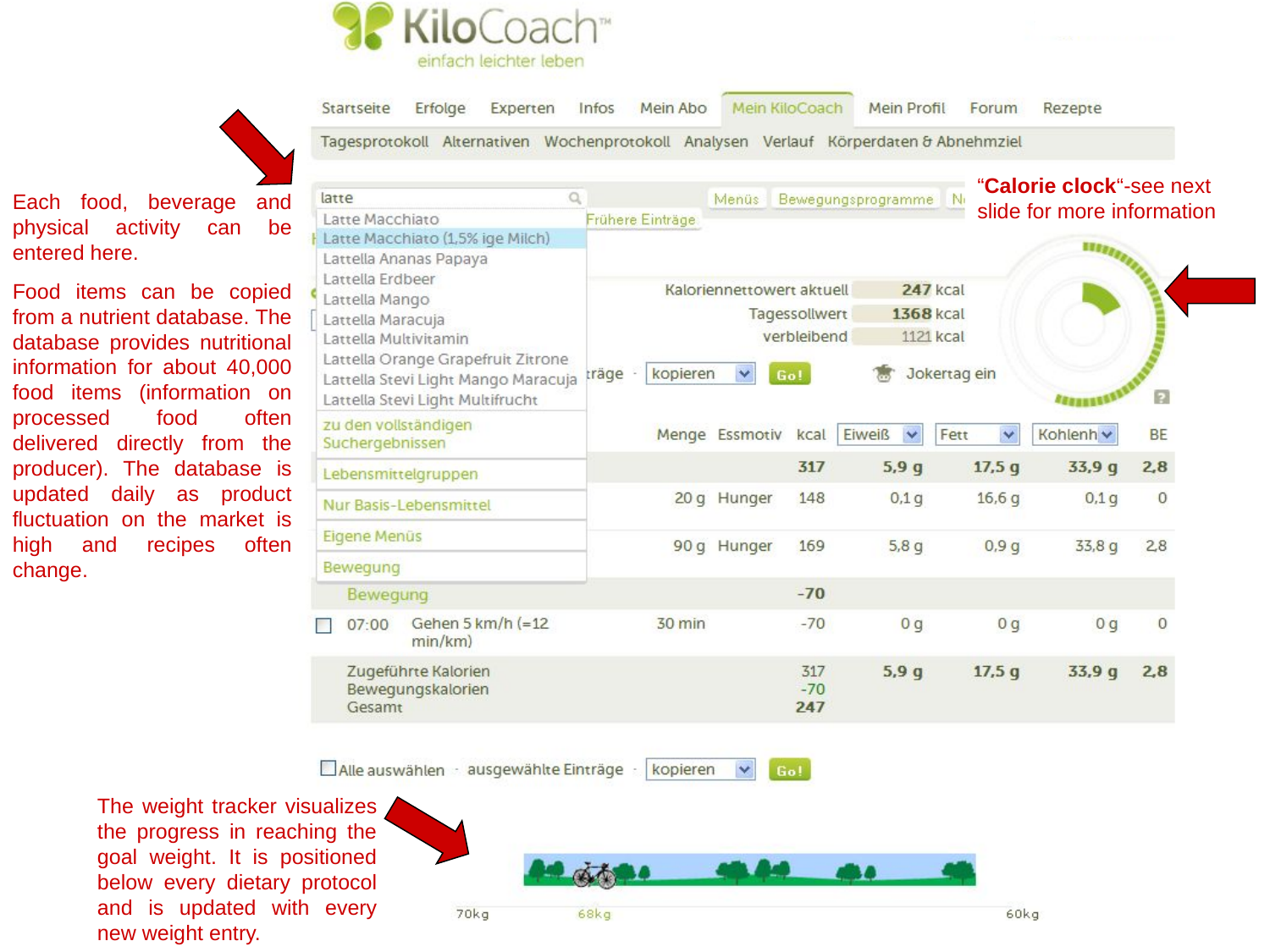

“Calorie clock“-see next slide for more information
Each food, beverage and physical activity can be entered here.
Food items can be copied from a nutrient database. The database provides nutritional information for about 40,000 food items (information on processed food often delivered directly from the producer). The database is updated daily as product fluctuation on the market is high and recipes often change.
The weight tracker visualizes the progress in reaching the goal weight. It is positioned below every dietary protocol and is updated with every new weight entry.

## Slide 3
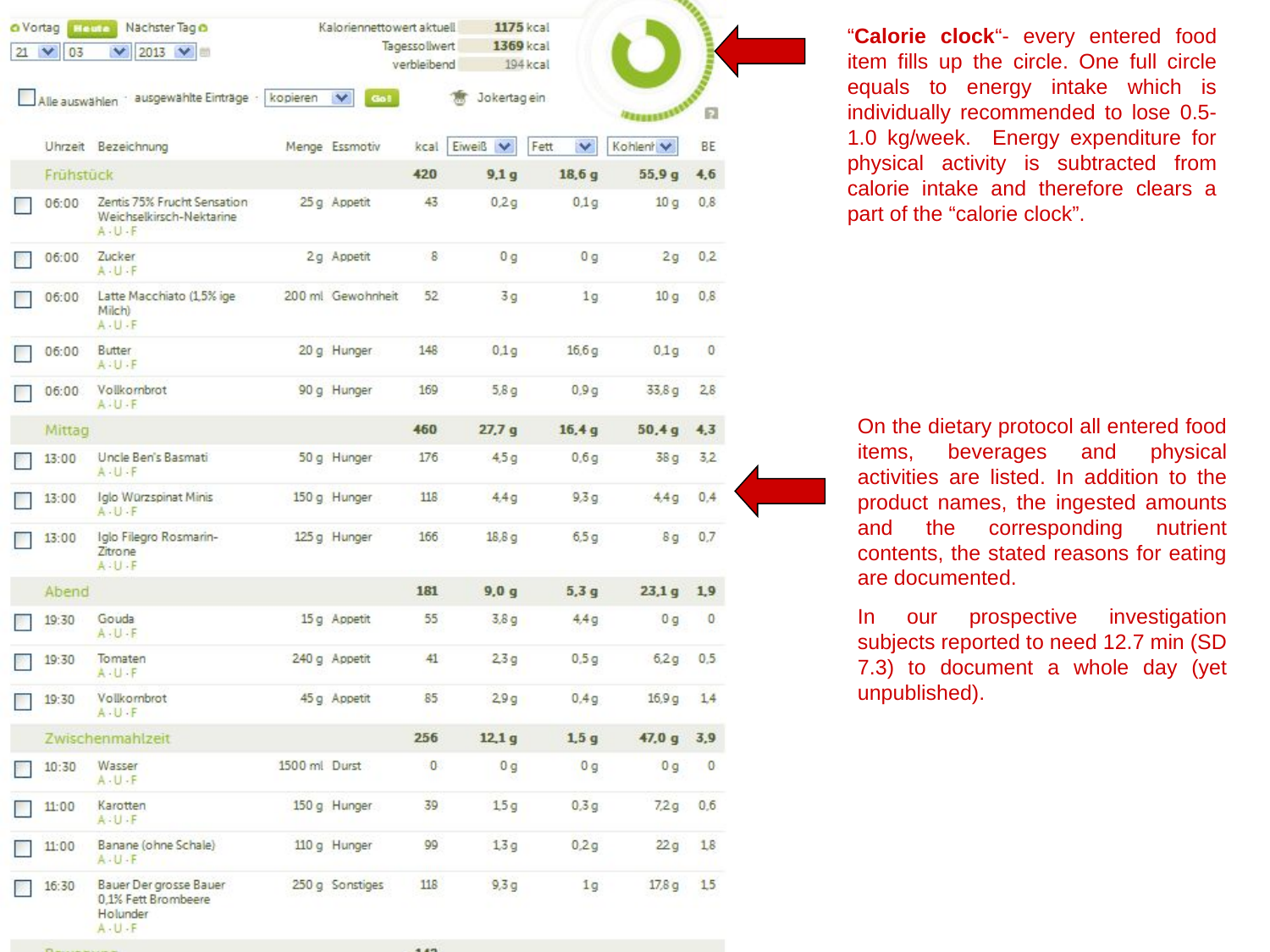

“Calorie clock“- every entered food item fills up the circle. One full circle equals to energy intake which is individually recommended to lose 0.5-1.0 kg/week. Energy expenditure for physical activity is subtracted from calorie intake and therefore clears a part of the “calorie clock”.
On the dietary protocol all entered food items, beverages and physical activities are listed. In addition to the product names, the ingested amounts and the corresponding nutrient contents, the stated reasons for eating are documented.
In our prospective investigation subjects reported to need 12.7 min (SD 7.3) to document a whole day (yet unpublished).

## Slide 4
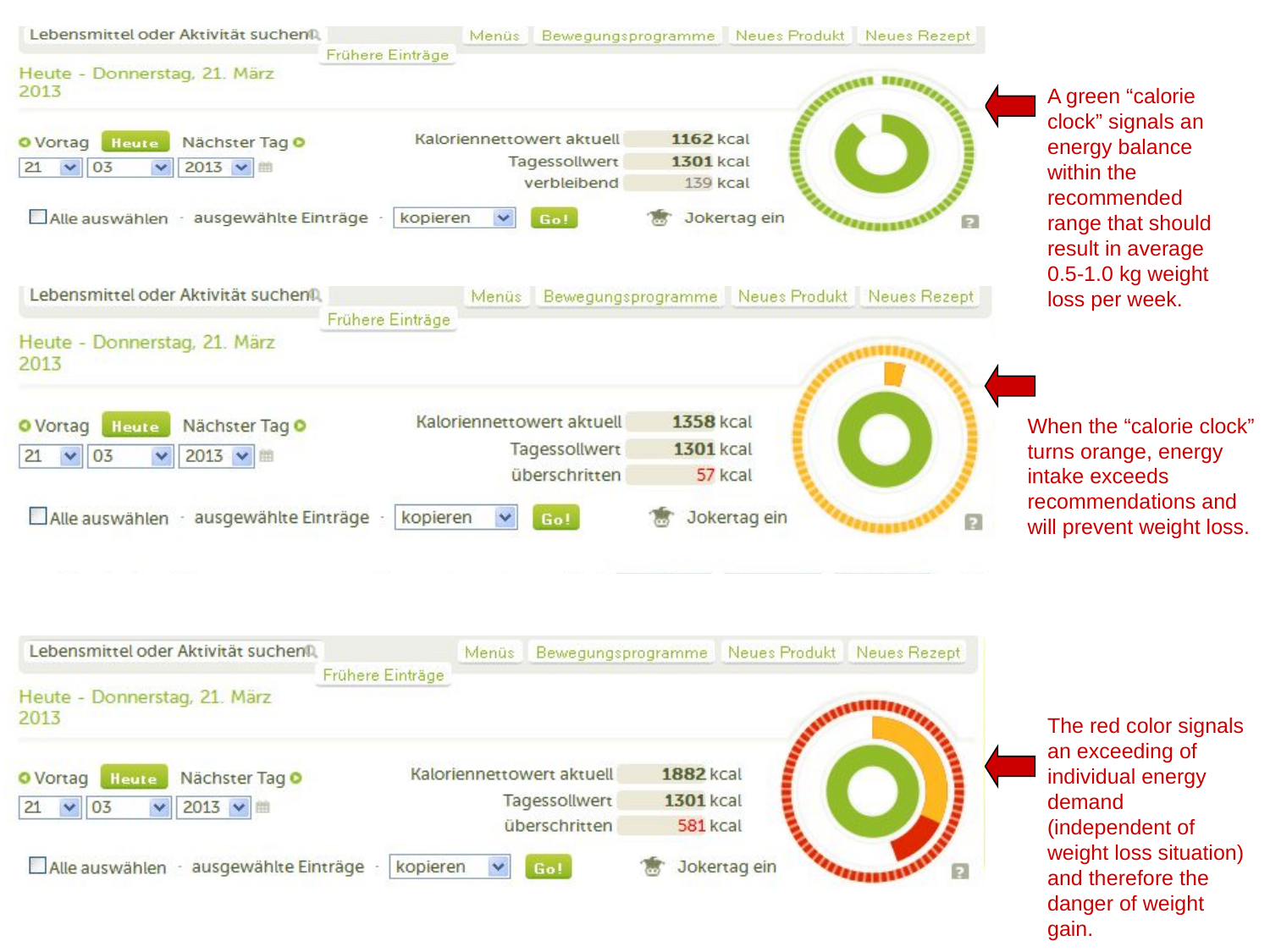

A green “calorie clock” signals an energy balance within the recommended range that should result in average 0.5-1.0 kg weight loss per week.
When the “calorie clock” turns orange, energy intake exceeds recommendations and will prevent weight loss.
The red color signals an exceeding of individual energy demand (independent of weight loss situation) and therefore the danger of weight gain.

## Slide 5
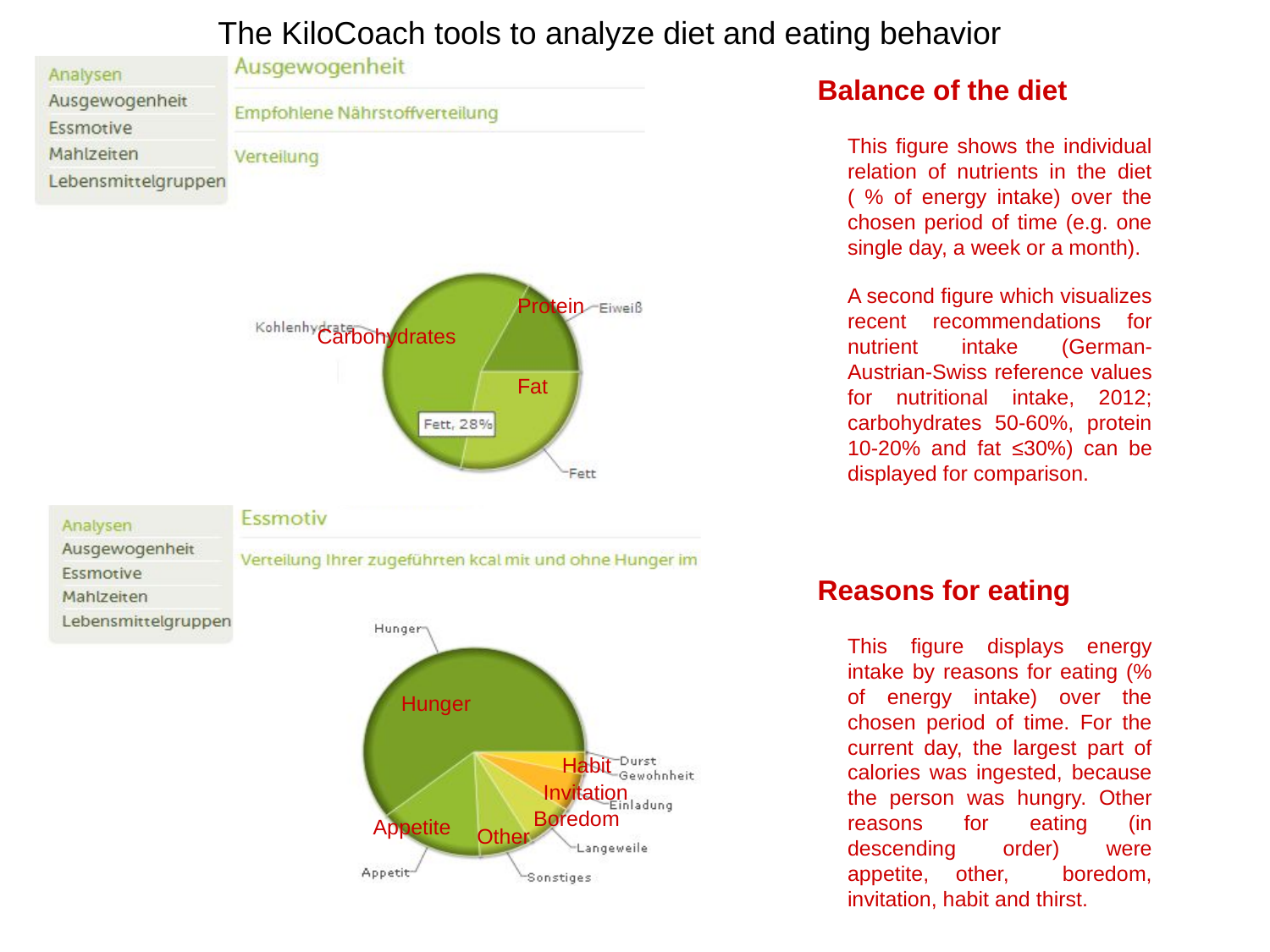

The KiloCoach tools to analyze diet and eating behavior
Protein
Carbohydrates
Fat
Balance of the diet
This figure shows the individual relation of nutrients in the diet ( % of energy intake) over the chosen period of time (e.g. one single day, a week or a month).
A second figure which visualizes recent recommendations for nutrient intake (German-Austrian-Swiss reference values for nutritional intake, 2012; carbohydrates 50-60%, protein 10-20% and fat ≤30%) can be displayed for comparison.
Hunger
Habit
Invitation
Boredom
Appetite
Other
Reasons for eating
This figure displays energy intake by reasons for eating (% of energy intake) over the chosen period of time. For the current day, the largest part of calories was ingested, because the person was hungry. Other reasons for eating (in descending order) were appetite, other, boredom, invitation, habit and thirst.

## Slide 6
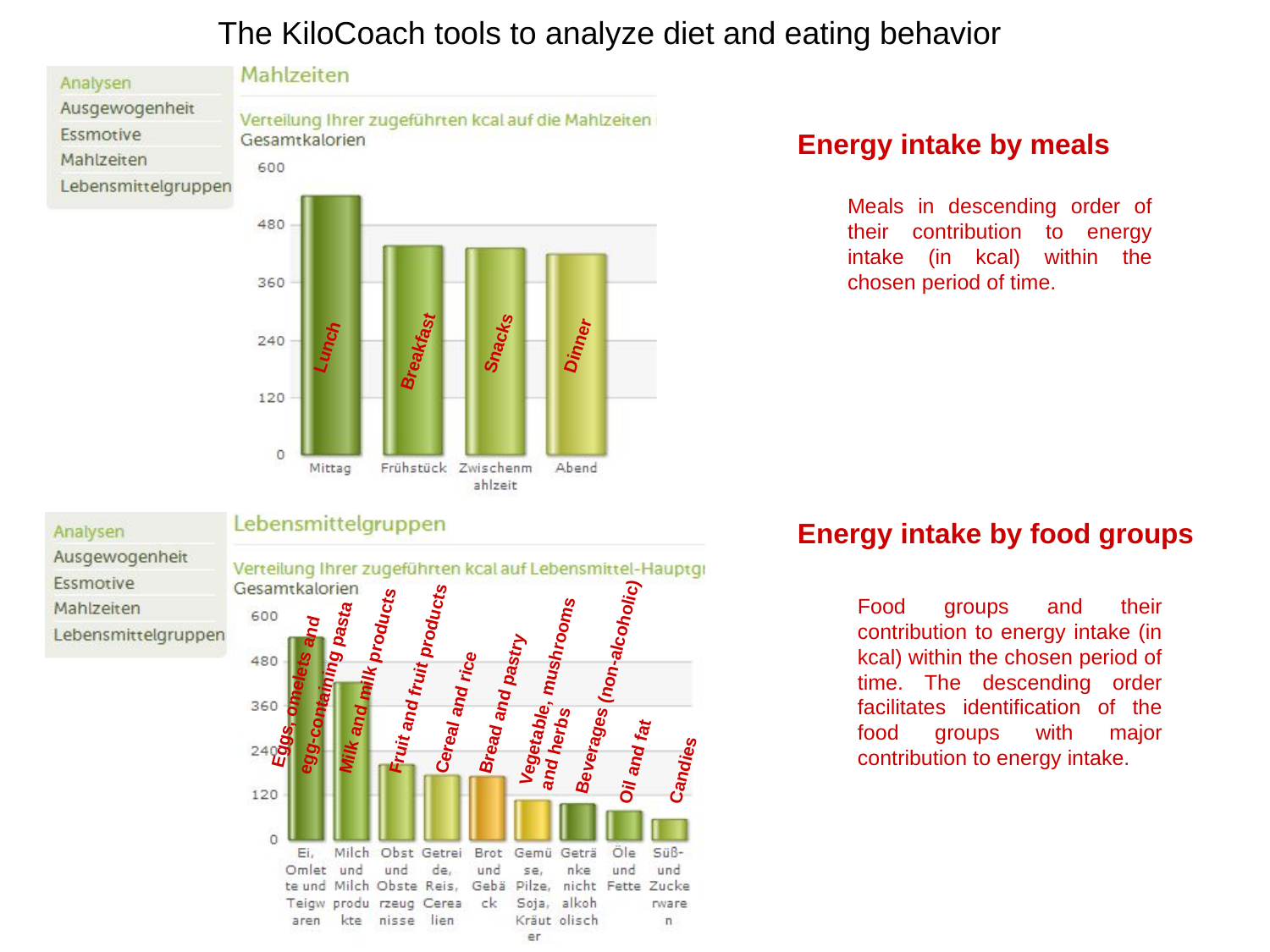

The KiloCoach tools to analyze diet and eating behavior
Lunch
Dinner
Snacks
Breakfast
Energy intake by meals
Meals in descending order of their contribution to energy intake (in kcal) within the chosen period of time.
Energy intake by food groups
Eggs, omelets and
egg-containing pasta
Fruit and fruit products
Vegetable, mushrooms and herbs
Milk and milk products
Beverages (non-alcoholic)
Bread and pastry
Cereal and rice
Oil and fat
Candies
Food groups and their contribution to energy intake (in kcal) within the chosen period of time. The descending order facilitates identification of the food groups with major contribution to energy intake.
