# Supplementary material for: Determinants of Successful Weight Loss After Using a Commercial Web-Based Weight Reduction Program for Six Months: Cohort Study
Source: J Med Internet Res. 2013 Oct 14;15(10):e219. doi: 10.2196/jmir.2648 (PMC3806515; doi:10.2196/jmir.2648)
Supplement: Supplementary file 2 [file jmir_v15i10e219_app2.pdf]

Table 1 Primary analysis: Percentage weight loss in the success groups unsuccessful (lost <5% of initial body weight), moderate success (lost 5%-9.9% of initial body weight), and high success (lost ≥10% initial body weight) from weeks 1-2 to weeks 25-26. Missing values were imputed using the last observation carried forward method.

	**Weeks 1-2**	**Weeks 3-4**	**Weeks 5-6**	**Weeks 7-8**	**Weeks 9-10**	**Weeks 11-12**	**Weeks 13-14**	**Weeks 15-16**	**Weeks 17-18**	**Weeks 19-20**	**Weeks 21-22**	**Weeks 23-24**	**Weeks 25-26**
**Unsuccessful**													
N	261	261	261	261	261	261	261	261	261	261	261	261	261
Missing N (%)	0 (0)	96 (36.8)	127 (48.7)	150 (57.5)	159 (60.9)	164 (62.8)	180 (69.0)	171 (65.5)	168 (64.4)	172 (65.9)	178 (68.2)	188 (72.0)	182 (69.7)
	0 (0)	84 (32.2)		131 (50.2)		142 (54.4)		141 (54.0)		138 (52.9)		149 (57.1)	
Mean (SD)	-0.3 (0.6)	-1.1(1,2)	-1.3 (1.5)	-1.5 (1.8)	-1.6 (2.0)	-1.7 (2.1)	-1.7 (2.2)	-1.7 (2.3)	-1.6 (2.5)	-1.6 (2.5)	-1.5 (2.4)	-1.3 (2.4)	-1.2 (2.4)
Median	-0.2	-1,0	-1.2	-1.2	-1.3	-1.5	-1.5	-1.6	-1.5	-1.6	-1.6	-1.3	-1.3
Min/Max	0.9/-2.0	1.8/-4.5	1.3/-5.9	7.1/-7.4	7.1/-8.7	7.1/-10.6	7.1/-10.8	8.0/-14.9	6.2/-14.9	6.4/-14.9	7.2/-9.7	7.2/-8.6	7.2/-5.0
**Moderate success**													
N	133	133	133	133	133	133	133	133	133	133	133	133	133
Missing N (%)	0 (0)	7 (5.3)	10 (7.5)	16 (12.0)	15 (11.3)	18 (13.5)	30 (22.6)	44 (33.1)	41 (30.8)	44 (33.1)	54 (40.6)	52 (39.1)	62 (46.6)
	0 (0)	4 (3.0)		6 (4.5)		11 (8.3)		26 (19.6)		32 (24.1)		38 (28.6)	
Mean (SD)	-0.7(0.7)	-2.5 (1.5)	-3.7 (1.7)	-4.6 (2.0)	-5.1 (2.0)	-5.7 (2.1)	-6.1 (2.1)	-6.3 (2.1)	-6.5 (2.0)	-6.7 (2.0)	-6.9 (1.8)	-7.2 (1.6)	-7.4 (1.5)
Median	-0.6	-2.6	-3.6	-4.6	-5.1	-5.5	-6.1	-6.6	-6.9	-6.9	-7.1	-7.2	-7.5
Min/Max	0.8/-2.3	0.7/-8.5	0.7/-7.8	0.7/-8.6	0.4/-9.2	0.9/-10.2	1.6/-10.2	0.9/-10.2	3.3/-10.6	3.3/-11.0	-0.5/-10.5	-2.2/-10.3	-5.0/-10.0
**High success**													
N	85	85	85	85	85	85	85	85	85	85	85	85	85
Missing N (%)	0 (0)	7 (8.2)	3 (3.5)	7 (8.2)	6 (7.1)	5 (5.9)	4 (4.7)	6 (7.1)	15 (17.7)	14 (16.5)	16 (18.8)	16 (18.8)	21 (24.7)
	0 (0)	2 (2.4)		4 (4.7)		2 (2.4)		4 (4.7)		9 (10.6)		12 (14.1)	
Mean (SD)	-0.9 (0.7)	-3.2 (1.5)	-4.9 (2.2)	-6.3 (2.7)	-7.6 (3.0)	-9.0 (3.3)	-10.1 (3.4)	-11.4 (3.3)	-12.0 (3.5)	-12.6 (3.6)	-13.2 (3.7)	-13.7 (3.9)	-14.2 (3.8)
Median	-0.9	-3.5	-4.9	-6.1	-7.4	-8.6	-9.7	-10.7	-11.6	-11.9	-12.2	-12.8	-13.1
Min/Max	0.9/-2.4	0.9/-6.8	0.9/-9.0	0.9/-12.2	0.9/-15.2	0.4/-10.9	0.0/-18.5	-2.0/-19.8	-2.8/-20.8	-2.8/-22.2	-2.8/-23.6	-2.8/-24.8	-10.1/-25.6
P [a]	**<.001**	**<.001**	**<.001**	**<.001**	**<.001**	**<.001**	**<.001**	**<.001**	**<.001**	**<.001**	**<.001**	**<.001**	**<.001**

[a] Kruskal-Wallis 1-way ANOVA over the 3 subgroups.

Table 2 Primary analysis: Body weight [kg] in the success groups unsuccessful (lost <5% of initial body weight), moderate success (lost 5%-9.9% of initial body weight), and high success (lost ≥10% initial body weight) during weight loss period from weeks 1-2 to weeks 25-26. Missing values were imputed using the last observation carried forward method.

	**Weeks 1-2**	**Weeks 3-4**	**Weeks 5-6**	**Weeks 7-8**	**Weeks 9-10**	**Weeks 11-12**	**Weeks 13-14**	**Weeks 15-16**	**Weeks 17-18**	**Weeks 19-20**	**Weeks 21-22**	**Weeks 23-24**	**Weeks 25-26**
**Unsuccessful**													
N	261	261	261	261	261	261	261	261	261	261	261	261	261
Missing N (%)	0 (0)	96 (36.8)	127 (48.7)	150 (57.5)	159 (60.9)	164 (62.8)	180 (69.0)	171 (65.5)	168 (64.4)	172 (65.9)	178 (68.2)	188 (72.0)	182 (69.7)
	0 (0)	84 (32.2)		131 (50.2)		142 (54.4)		141 (54.0)		138 (52.9)		149 (57.1)	
Mean (SD)	94.8 (14.9)	94.1 (14.7)	93.8 (14.7)	93.7 (14.7)	93.6 (14.7)	93.5 (14.7)	93.5 (14.6)	93.5 (14.6)	93.5 (14.6)	93.5 (14.6)	93.7 (14.6)	93.9 (14.7)	94.0 (14.7)
Median	95.0	94.1	94.0	94.0	93.9	94.0	93.9	93.8	93.9	93.6	93.6	93.6	93.7
Min/Max	63.0/142.5	63.0/142.5	63.0/142.5	63.0/142.5	63.0/142.5	63.0/142.5	63.0/142.5	63.0/142.5	60.0/142.5	60.0/142.5	60.0/142.5	60.0/142.5	60.0/142.5
**Moderate success**													
N	133	133	133	133	133	133	133	133	133	133	133	133	133
Missing N (%)	0 (0)	7 (5.3)	10 (7.5)	16 (12.0)	15 (11.3)	18 (13.5)	30 (22.6)	44 (33.1)	41 (30.8)	44 (33.1)	54 (40.6)	52 (39.1)	62 (46.6)
	0 (0)	4 (3.0)		6 (4.5)		11 (8.3)		26 (19.6)		32 (24.1)		38 (28.6)	
Mean (SD)	92.0 (13.5)	90.3 (13.2)	89.3 (12.9)	88.4 (12.7)	87.9 (12.6)	87.4 (12.5)	87.0 (12.4)	86.8 (12.3)	86.6 (12.3)	86.4 (12.4)	86.2 (12.3)	86.0 (12.3)	85.8 (12.3)
Median	89.5	88.3	87.1	86.2	86.0	85.4	85.0	84.7	84.5	84.3	84.3	84.2	84.1
Min/Max	68.6/134.5	68.2/130.9	67.3/129.0	66.8/127.2	67.0/127.0	66.4/127.0	66.2/125.5	65.9/124.8	66.0/124.8	65.4/124.8	64.9/124.8	65.2/124.8	65.4/124.8
**High success**													
N	85	85	85	85	85	85	85	85	85	85	85	85	85
Missing N (%)	0 (0)	7 (8.2)	3 (3.5)	7 (8.2)	6 (7.1)	5 (5.9)	4 (4.7)	6 (7.1)	15 (17.7)	14 (16.5)	16 (18.8)	16 (18.8)	21 (24.7)
	0 (0)	2 (2.4)		4 (4.7)		2 (2.4)		4 (4.7)		9 (10.6)		12 (14.1)	
Mean (SD)	93.5 (13.9)	91.3 (13.6)	89.6 (13.2)	88.3 (13.0)	87.1 (12.7)	85.7 (12.5)	84.6 (12.3)	83.5 (12.1)	82.8 (11.9)	82.3 (11.9)	81.7 (11.7)	81.3 (11.7)	80.7 (11.7)
Median	93.0	89.5	88.7	86.5	86.0	85.0	84.3	83.7	82.3	81.5	81.2	80.9	80.0
Min/Max	69.7/132.8	69.3/129.6	68.1/127.5	66.6/126.8	65.4/123.8	64.6/121.5	64.0/120.2	62.8/119.0	62.9/117.4	63.1/115.8	63.1/115.0	63.2/114.3	62.0/113.8
P [a]	**.16**	**.02**	**.003**	**<.001**	**<.001**	**<.001**	**<.001**	**<.001**	**<.001**	**<.001**	**<.001**	**<.001**	**<.001**

[a] Kruskal-Wallis 1-way ANOVA over the 3 subgroups.

Table 3 Percentage weight loss in the success groups unsuccessful (lost <5% of initial body weight), moderate success (lost 5%-9.9% of initial body weight), and high success (lost ≥10% initial body weight) in the follow-up period.

	After 1 year	After 1.5 years	After 2 years
**Unsuccessful**			
N	74	44	34
Mean (SD)	-1.6 (3.7)	-0.9 (4.3)	-0.8 (4.8)
Median	-1.1	-0.4	-0.4
Min/Max	3.7/-15.7	9.0/-12.9	7.6/-14.9
**Moderate success**			
N	66	38	28
Mean (SD)	-6.3 (4.9)	-4.5 (4.4)	-3.9 (4.6)
Median	-6.7	-5.3	-3.9
Min/Max	10.0/-16.1	4.4/-11.8	6.6/-18.7
**High success**			
N	58	45	28
Mean (SD)	-13.5 (6.7)	-12.4 (7.7)	-11.2 (8.9)
Median	-12.9	-10.9	-9.3
Min/Max	-1.75/-28.2	0.9/-27.7	3.4/-32.9
P [a]	**< .001**	**< .001**	**< .001**

Table 4 Body weight in kg in the success groups unsuccessful (lost <5% of initial body weight), moderate success (lost 5% -9.9% of initial body weight), and high success (lost ≥10% initial body weight) in the follow-up period.

	After 1 year	After 1.5 years	After 2 years
**Unsuccessful**			
N	74	44	34
Mean (SD)	94.9 (15.7)	95.8 (13.4)	96.2 (12.6)
Median	96.5	97.9	97.5
Min/Max	62.0/129.7	66/120.7	69.0/120.5
**Moderate success**			
N	66	38	28
Mean (SD)	86.3 (13.1)	91.4 (15.3)	91.1 (15.4)
Median	82.6	91.2	92.1
Min/Max	77.3/118.5	65.2/119.0	66.7/121.9
**High success**			
N	58	45	28
Mean (SD)	82.6 (14.5)	81.4 (12.0)	81.5 (15.5)
Median	81.2	81.4	77.6
Min/Max	60.8/122.8	60.8/115.0	59.8/120.2
P [a]	**< .001**	**< .001**	**.001**

[a] Kruskal-Wallis 1-way ANOVA over the 3 subgroups.

Table 5 Program usage intensity (and energy intake) within the 6-month weight loss period are given as mean (SD): unsuccessful (lost <5% of initial body weight), moderate success (lost 5%-9.9% of initial body weight), and high success (lost ≥10% initial body weight).

	Unsuccessful	Moderate success	High success	*P* [a]
Dietary protocols / week [n]	2.4 (2.2)	4.6 (1.8)	5.3 (1.7)	< .001
Weight entries / week [n]	0.6 (0.8)	1.2 (1.0)	1.7 (1.2)	< .001
Logbook entries / week [n]	0.7 (1.0)	1.6 (1.5)	2.1 (1.7)	< .001
Meals / day [n]	2.9 (0.6)	3.2 (0.5)	3.2 (0.6)	< .001
Energy intake / day [kcal]	1704 (607)	1984 (675)	2156 (740)	< .001

[a] Kruskal-Wallis-one-way analysis of variance over the three subgroups.
