# Supplementary material for: Determinants of Successful Weight Loss After Using a Commercial Web-Based Weight Reduction Program for Six Months: Cohort Study
Source: J Med Internet Res. 2013 Oct 14;15(10):e219. doi: 10.2196/jmir.2648 (PMC3806515; doi:10.2196/jmir.2648)
Supplement: Supplementary file 3 [file jmir_v15i10e219_app3.pdf]

Table 1 Sensitivity analysis – baseline carried forward method: Percentage weight loss in the success groups unsuccessful (lost <5% of initial body weight), moderate success (lost 5%-9.9% of initial body weight), and high success (lost ≥10% initial body weight) from weeks 1-2 to weeks 25-26. Missing weight data were imputed using the baseline carried forward method.

	Weeks 1-2	Weeks 3-4	Weeks 5-6	Weeks 7-8	Weeks 9-10	Weeks 11-12	Weeks 13-14	Weeks 15-16	Weeks 17-18	Weeks 19-20	Weeks 21-22	Weeks 23-24	Weeks 25-26
**Unsuccessful**													
N	344	344	344	344	344	344	344	344	344	344	344	344	344
Missing N (%)	0 (0)	104 (30.2)	137 (39.8)	165 (48.0)	173 (50.3)	184 (53.5)	208 (60.5)	207 (60.2)	203 (59.0)	215 (62.5)	224 (65.1)	238 (69.2)	265 (77.0)
	0 (0)	88 (25.6)		138 (40.1)		155 (45.1)		162 (47.1)		169 (49.1)		199 (57.9)	
Mean (SD)	-0.5 (0.6)	-1.4 (1.5)	-1.8 (2.1)	-1.9 (2.7)	-2.0 (3.0)	-2.1 (3.2)	-1.9 (3.4)	-1.9 (3.6)	-1.6 (3.3)	-1.6 (3.5)	-1.4 (3.3)	-1.2 (3.0)	-0.4 (1.4)
Median	-0.3	-1.2	-0.9	0.0	0.0	0.0	0.0	0.0	0.0	0.0	0.0	0.0	0.0
Min/Max	0.9/-2.3	1.8/-6.3	1.3/-8.8	7.1/-12.2	2.8/-15.2	3.2/-16.7	6.2/-18.5	8.0/-19.8	5.7/-17.2	4.8/-20.7	7.2/-21.3	5.7/-17.9	5.73/-5.0
**Moderate success**													
N	71	71	71	71	71	71	71	71	71	71	71	71	71
Missing N (%)	0 (0)	3 (4.2)	1 (1.4)	5 (7.0)	4 (4.6)	2 (2.8)	6 (8.5)	11 (15.5)	15 (21.1)	8 (11.3)	18 (25.4)	14 (19.7)	0 (0)
	0 (0)	1 (1.4)		2 (2.8)		0 (0)		8 (11.3)		7 (9.9)		0 (0)	
Mean (SD)	-0.7 (0.7)	-2.4 (1.5)	-3.6 (1.6)	-4.3 (2.0)	-4.7 (2.1)	-5.5 (2.0)	-5.6 (2.3)	-5.5 (2.8)	-5.4 (3.2)	-6.2 (2.7)	- 5.4 (3.5)	-6.0 (3.3)	-7.6 (1.5)
Median	-0.7	-2.4	-3.4	-4.1	-4.7	-5.4	-5.9	-6.3	-6.5	-6.9	-6.3	-6.7	-7.8
Min/Max	0.8/-2.2	0.6/-8.5	0.0/-7.8	0.0/-8.4	0.0/-9.2	0.0/-10.2	0.0/-9.3	0.0/-9.5	0.0/-10.6	0.0/-11.0	0.0/-10.5	0.0/-10.3	-5.0/-10.0
**High success**													
N	64	64	64	64	64	64	64	64	64	64	64	64	64
Missing N (%)	0 (0)	3 (4.7)	2 (3.1)	3 (4.7)	3 (4.7)	1 (1.6)	0 (0)	3 (4.7)	6 (9.4)	7 (10.9)	6 (9.4)	4 (6.3)	0 (0)
	0 (0)	1 (1.6)		1 (1.6)		0 (0)		1 (1.6)		3 (4.7)		0 (0)	
Mean (SD)	-0.9 (0.7)	-3.4 (1.3)	-5.0 (2.1)	-6.4 (2.4)	-7.4 (2.9)	-8.9 (3.1)	-10.0 (3.2)	-10.7 (4.1)	-10.9 (5.0)	-11.2 (5.4)	-12.2 (5.4)	-13.0 (5.1)	-11.9 (3.6)
Median	-0.9	-3.6	-5.0	-6.4	-7.3	-8.5	-9.4	-10.6	-11.3	-11.1	-12.0	-12.6	-13.5
Min/Max	0.9/-2.4	0.0/-6.8	0.0/-9.0	0.0/-11.6	0.0/-13.4	0.0/-16.5	0.0/-18.0	0.0/-19.3	0.0/-20.8	0.0/-22.2	0.0/-23.6	0.0/-24.8	-10.1/-25.6
P [a]	**<.001**	**<.001**	**<.001**	**<.001**	**<.001**	**<.001**	**<.001**	**<.001**	**<.001**	**<.001**	**<.001**	**<.001**	**<.001**

[a] Kruskal-Wallis 1-way ANOVA over the 3 subgroups.

Table 2 Sensitivity analysis - subgroup evaluation: Percentage weight loss in the success groups unsuccessful (lost <5% of initial body weight), moderate success (lost 5%-9.9% of initial body weight), and high success (lost ≥10% initial body weight) from weeks 1-2 to weeks 25-26 in users who entered a weight in weeks 25-26. Missing weight data within this period were imputed using the last observation carried forward method.

	Weeks 1-2	Weeks 3-4	Weeks 5-6	Weeks 7-8	Weeks 9-10	Weeks 11-12	Weeks 13-14	Weeks 15-16	Weeks 17-18	Weeks 19-20	Weeks 21-22	Weeks 23-24	Weeks 25-26
**Unsuccessful**													
N	79	79	79	79	79	79	79	79	79	79	79	79	79
Missing N (%)	0 (0)	19 (24.1)	27 (34.2)	33 (41.8)	35 (44.3)	35 (44.3)	44 (55.7)	34 (43.0)	29 (36.7)	34 (43.0)	39 (49.4)	39 (49.4)	0 (0)
	0 (0)	18 (22.8)		27 (34.2)		31 (39.2)		23 (29.1)		22 (27.9)		0 (0)	
Mean (SD)	-0.4 (0.6)	-1.0 (1.1)	-1.5 (1.5)	-1.7 (1.9)	-1.9 (2.1)	-2.1 (2.2)	-2.1 (2.4)	-2.1 (2.4)	-2.3 (2.7)	-2.4 (2.5)	-2.2 (2.6)	-1.9 (2.6)	-1.6 (2.6)
Median	-0.3	-1.0	-1.3	-1.7	-2.0	-2.3	-2.2	-2.4	-2.4	-2.4	-2.1	-2.2	-2.0
Min/Max	0.9/-2.1	1.8/-3.4	1.1/-5.3	2.5/-5.8	2.7/-6.4	2.8/-7.3	3.6/-6.9	4.1/-6.4	5.7/-12.4	3.0/-9.7	3.9/-9.7	5.7/-8.6	5.7/-5.0
**Moderate success**													
N	71	71	71	71	71	71	71	71	71	71	71	71	71
Missing N (%)	0 (0)	3 (4.2)	1 (1.4)	5 (7.0)	4 (4.6)	2 (2.8)	6 (8.5)	11 (15.5)	15 (21.1)	8 (11.3)	18 (25.4)	14 (19.7)	0 (0)
	0 (0)	1 (1.4)		2 (2.8)		0 (0)		8 (11.3)		7 (9.9)		0 (0)	
Mean (SD)	-0.7 (0.7)	-2.5 (1.5)	-3.6 (1.6)	-4.5 (1.8)	-4.9 (1.9)	-5.6 (1.8)	-6.2 (1.7)	-6.5 (1.7)	-6.7 (1.8)	-6.9 (1.8)	-7.1 (1.8)	-7.2 (1.7)	-7.6 (1.5)
Median	-0.7	-2.4	-3.4	-4.1	-4.9	-5.4	-6.2	-6.6	-7.0	-7.1	-7.2	-7.5	-7.8
Min/Max	0.8/-2.2	0.6/-8.5	0.0/-7.8	0.0/-8.4	0.0-/9.2	0.0/-10.2	-2.6/-10.2	-2.6/-10.2	-2.6/-10.6	-2.6/-11.0	-2.6/-10.5	-2.2/-10.3	-5.0/-10.0
**High success**													
N	64	64	64	64	64	64	64	64	64	64	64	64	64
Missing N (%)	0 (0)	3 (4.7)	2 (3.1)	3 (4.7)	3 (4.7)	1 (1.6)	0 (0)	3 (4.7)	6 (9.4)	7 (10.9)	6 (9.4)	4 (6.3)	0 (0)
	0 (0)	1 (1.6)		1 (1.6)		0 (0)		1 (1.6)		3 (4.7)		0 (0)	
Mean (SD)	-0.9 (0.7)	-3.4 (1.4)	-5.0 (2.0)	-6.4 (2.3)	-7.6 (2.7)	-9.1(2.9)	-10.0 (3.2)	-11.2 (3.4)	-11.9 (3.6)	-12.4 (3.8)	-13.1 (4.0)	-13.7 (4.1)	-11.9 (3.6)
Median	-0.9	-3.6	-5.0	-6.4	-7.4	-8.6	-9.4	-10.6	-11.6	-11.7	-12.2	-12.9	-13.5
Min/Max	0.9/-2.4	0.9/-6.8	0.9/-9.0	0.9/-11.6	0.9/-13.4	-1.7/-16.5	0.0/-18.0	-2.0/-19.3	-2.8/-20.8	-2.8/-22.2	-2.8/-23.6	-2.8/-24.8	-10.1/-25.6
P [a]	**<.001**	**<.001**	**<.001**	**<.001**	**<.001**	**<.001**	**<.001**	**<.001**	**<.001**	**<.001**	**<.001**	**<.001**	**<.001**

[a] Kruskal-Wallis 1-way ANOVA over the 3 subgroups.
